# Case report: cranioplasty infection due to Roseomonas gilardii at a university hospital in turkey

**DOI:** 10.11604/pamj.2013.14.162.2730

**Published:** 2013-04-25

**Authors:** Gulfem Ece, Mete Ruksen, Ali Akay

**Affiliations:** 1 Izmir University School of Medicine Medicalpark Hospital Department of Medical Microbiology; 2 Izmir University School of Medicine Medicalpark Hospital Department of Neurosurgery

**Keywords:** R. gilardii, cranioplasty, pink-pigmented bacilli

## Abstract

Roseomonas is a pink-pigmented, nonfermentative, oxidative, Gram-negative coccobacilli that has clinical importance as opportunistic pathogen which can lead to infections especially in immunosuppressed individuals. It is relatively less reported in many centers. These microorganisms are detected after several days growth in culture environment, and typical pink, mucoid colonies are detected. We are reported a case of cranioplasty infection that took place in a patient with with cranial abscess formation due to Roseomonas gilardii at Izmir University School of Medicine Medicalpark Hospital.

## Introduction

Gilardi and Faur showed a new group of pink-pigmented nonfermentative bacteria and then it was defined by the Centers for Disease Control and Prevention to indicate these bacteriae in 1984 [1]. *Roseomonas* is a pink-pigmented, nonfermentative, oxidase (+), Gram-negative coccobacilli that has clinical importance as opportunistic bacteria which can lead to infections especially in immunosuppressed individuals. Most infections due to *Roseomonas* spp. are detected in patients with central venous catheters and underlying disorders. It is less reported in many hospitals because of having little experience in identification and less clinicians dealing with these infections [2, 3]. They are detected after several days of growth in culture environment, characteristic pink, mucoid colonies are observed [4].

Although some strains were isolated from environment, the natural source of *Roseomonas* spp. is still a challenge. The clinical specimens in which the microorganism was isolated include wounds, exudates, abscesses and genitourinary specimens. Besides, infection can be related with peritoneal dialysis and vertebral osteomyelitis. The clinical importance of these isolates is an essential issue in individuals with underlying disorders such as cancer and diabetes and in a study that reviewed of 35 cases from which *Roseomonas* strains were isolated, 60% were found to be related with disease [5].

We are reported a case of wound infection that took place in a patient with with cranial abscess formation due to *Roseomonas gilardii* at Izmir University School of Medicine Medicalpark Hospital.

## Patient and observation

**Figure 1:**
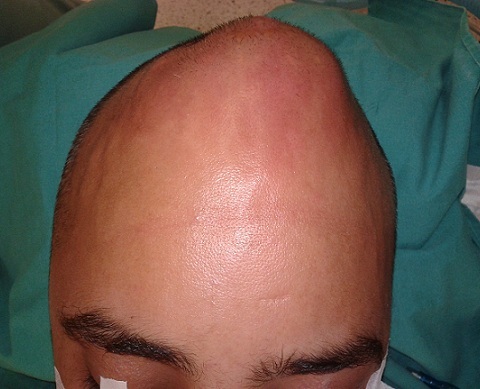
Abscess formation on the frontal bone

**Figure 2:**
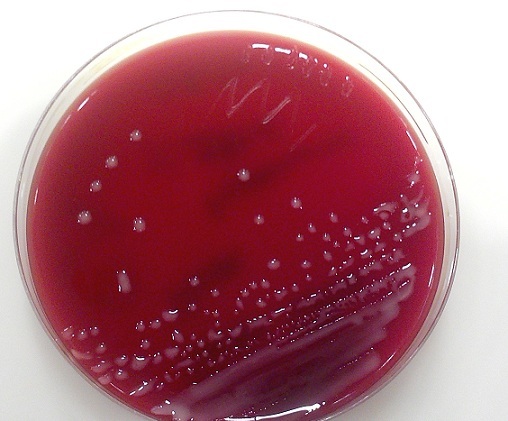
Roseomonas gliardii on blood agar (pink-mucoid colony)

**Figure 3:**
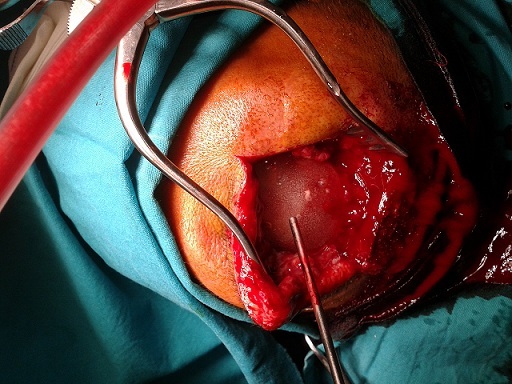
Removal of the cranioplasty

**Figure 4:**
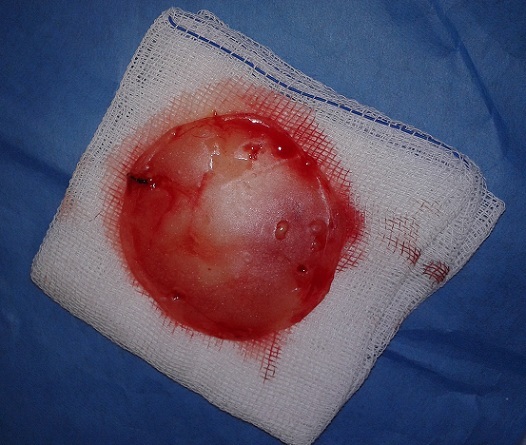
The cranioplasty

30-year old male patient had decompression operation due to cranial fracture 15 years ago. He had cranioplasty operation 12 years ago. Two weeks ago he complained of headache, and abscess formation on the frontal bone (Figure 1). The abscess formation was surgically drained at the Neurosurgey Department and sent to Izmir University School of Medicine Clinical Microbiology Laboratory. The specimen was cultivated on blood agar, chocolate agar and eosin metylene blue agar. After two days of incubation at 37°C; pink mucoid colonies were detected on blood agar. The identification of the strain was carried out by automatized Vitek 2.0 (Biomerieux, France). The culture reported the isolate as *Roseomonas gliardii* (Figure 2). The isolate was susceptible to amikacin, imipenem, levofloxacin, tigecycline, ciprofloxacin, and resistant to meropenem and tazobactam/piperacillin. Then his complaints continued and he underwent neurosurgical operation and cranioplasty was removed the from the cranium (Figure 3, Figure 4). The patient recovered thereafter.

## Discussion

*Roseomonas* is a Gram-negative bacilli that is known as pink-pigmented bacilli and classified in groups I through IV by Centers for Disease Control and Prevention [6]. Infection usually takes place in patients with underlying medical illness such as malignancy, acquired immune deficiency syndrome, chronic renal disease or diabetes mellitus. We reported a patient with *Roseomonas giliardii* infection after cranioplasty operation that took place twelve years ago.

De et al analyzed 36 cases of bacteremia or catheter-related infection caused by *Roseomonas* species, a group of pink, slimy, waterborne, Gram-negative coccobacilli.. Twenty nine (81%) of the cases were symptomatic, with fever being the most common symptom. Twenty (56%) of the infections were caused by a single microorganism. Six cases (17%) had catheter colonization, and five of them removed catheter to stop infection. All patients healed with empirical antibiotic treatment. The antibiotic susceptibility pattern of these strains and other reported series showed that *Roseomonas* species are susceptible to amikacin and imipenem and frequently susceptible to ciprofloxacin and ticarcillin, but essentially nonsusceptible to ceftazidime and cefepime. This result may facilitate treatment for infections due to *Roseomonas* species [7]. In our case similarly the isolate was susceptible to imipenem and amikacin; and resistant to ceftazidime.

Wang et al evaluated the database of the Bacteriology Laboratory at the National Taiwan University Hospital to identify patients with infections caused by *Roseomonas* species during a decade. Twenty patients had cultures positive for Roseomonas species. The authors concluded that *Roseomonas* species can cause infection in children and adults independent of immunity [8].

Nolan et al report the first case of ventriculitis caused by *R. Gilardii* in a 54-year-old man with a subarachnoid haemorrhage secondary to a vertebral artery aneurysm [3]. In our case report the patient underwent decompression operation and that may be the major risk factor.

Singal et al reported the first case of left ventricular assist device infection secondary to *Roseomonas*. The patient was a 48-year-old man with nonischemic cardiomyopathy and severe aortic regurgitation. He underwent implantation and during transplantion, a collection of granulation tissue was detected behind the ascending aorta. Drainage of this clinical specimen underwent culture and grew pink mucoid colonies and the culture reported as *Roseomonas* species. The isolate was resistant to trimethoprim-sulfamethoxazole, ceftazidime, and piperacillin-tazobactam and intermediate susceptibility to cefuroxime [9]. The isolate was susceptible to cefazolin, cefotetan, ceftriaxone, gentamicin, amikacin, doxycycline, ciprofloxacin, aztreonam, and meropenem. In our case report the isolate was susceptible to ciprofloxacin; but resistant to meropenem and there was a history of cranioplasty operation.

## Conclusion

In our case we reported a patient with *Roseomonas gilardii* infection cranioplasty operation that took place more than a decade ago. This case emphasizes the potential for organisms that cause infection indwelling catheters particularly in immunosuppressed ones and this should be kept in mind.
